# A Rare Case of Solitary Fibrous Tumor of the Kidney

**DOI:** 10.7759/cureus.46475

**Published:** 2023-10-04

**Authors:** Muhammad Tahir, Rosetta Campbell, Hannah Moreland, Carlina Madelaire, Christopher E Keel, Guillermo A Herrera

**Affiliations:** 1 Pathology and Laboratory Medicine, University of South Alabama Health Hospital, Mobile, USA; 2 Urology, University of South Alabama Health Hospital, Mobile, USA; 3 Pathology, College of Medicine, University of South Alabama Health Hospital, Mobile, USA

**Keywords:** sft, solitary fibrous tumor electron microscopy, solitary fibrous tumor of the kidney, renal neoplasm, solitary fibrous tumor (sft)

## Abstract

Solitary fibrous tumor (SFT), originally described in the pleura, is a rare mesenchymal neoplasm characterized by a wide spectrum of clinical presentations and histopathological features. Over the years, SFTs have been reported in various anatomical locations, including soft tissues, visceral organs, and, uncommonly, the kidney. While SFTs primarily arise from the pleura, their occurrence in the kidney is an infrequent phenomenon, accounting for a minute fraction of all renal tumors. This case report presents a unique instance of a solitary fibrous tumor originating in the kidney, highlighting its clinical, radiological, and histopathological characteristics, as well as the challenges associated with accurate diagnosis and appropriate management. The rarity of such cases underscores the importance of comprehensive evaluation and awareness among clinicians and pathologists to ensure timely diagnosis and effective treatment strategies. Here, we report a case of SF of the kidney (SFT-K) located in the renal pelvis in a 39-year-old Caucasian female.

## Introduction

Solitary fibrous tumors (SFTs) are a rare type of mesenchymal neoplasm, representing approximately 2% of all soft tissue tumors [1]. This soft tissue tumor was first identified in the pleura, but it can occur in various locations throughout the body, including the kidney. SFTs of the kidney (SFT-K) are rare, and there have been a total of 105 cases of SFT-K that have been reported in the literature [1, 2]. The clinical presentation of SFT-K can vary widely, ranging from asymptomatic incidental findings to abdominal discomfort, hematuria, renal mass, and large abdominal mass. Radiologic imaging aids in the initial assessment, but a definitive diagnosis relies on histopathological and immunohistochemical evaluation [3]. A few patients with extra-pleural SFT have been presented with hypoglycemia and paraneoplastic syndrome [4]. Histologically, these tumors are characterized by a unique histological appearance and the presence of fibroblast-like cells within a collagen-rich stroma. They also display a hemangiopericytoma-like growth pattern with immunohistochemical staining for CD34, Bcl-2, and STAT-6, which are considered diagnostic markers for those tumors. Surgical resection remains the cornerstone of treatment for localized SF T-K with favorable outcomes and is reported in most cases [2,3]. However, due to their severity, optimal management strategies and long-term follow-up plans are not well defined.

## Case presentation

**Figure 1:**
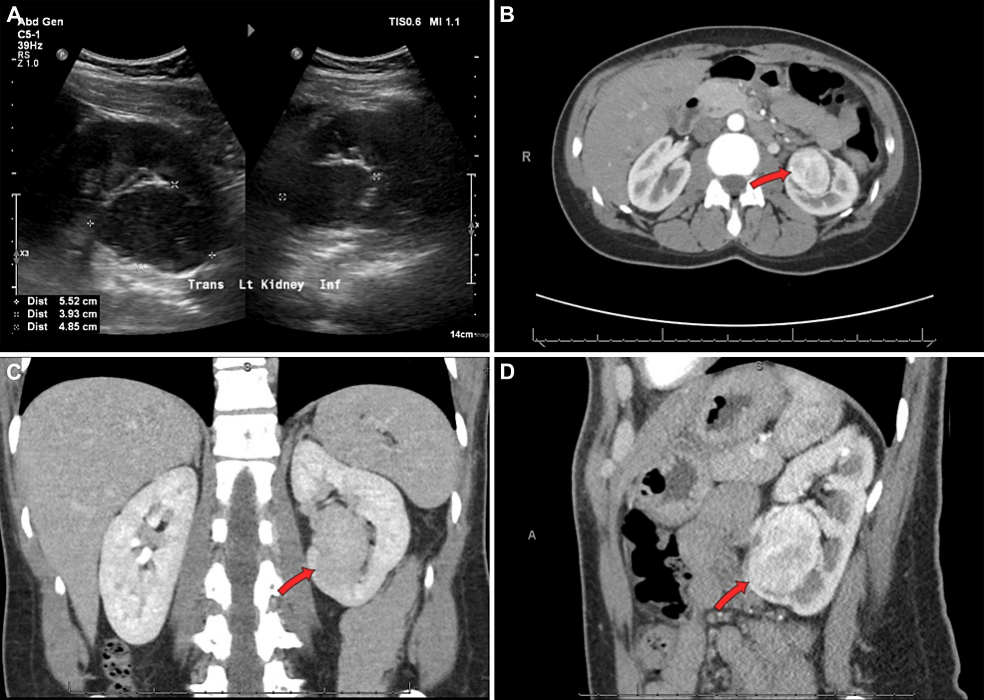
Round isoechoic solid left renal mass extending from the inferior pole (A); heterogeneously enhancing mass in the lower pole of the left kidney, coronal, axial, and sagittal views, respectively (B, C, D).

A 39-year-old Caucasian female with a past medical history significant for hypothyroidism presented to the hospital as a day of surgery admission for a left partial nephrectomy of a presumed renal cell carcinoma. The patient was in good health and denied any prior urological symptoms or history. About four months prior, she was seen by her primary care provider due to right flank pain, which prompted imaging. A CT scan was obtained that showed a heterogeneously enhancing mass in the lower pole of the left kidney, measuring 3.6 x 3.3 cm. The mass was noted to be highly suspicious for renal cell carcinoma (Figure 1). The initial surgical plan was for a partial nephrectomy; however, this was converted to a radical nephrectomy due to an intra-operative histological evaluation more consistent with a soft tissue tumor. Overall, she tolerated the surgery well, without any significant post-operative complications.

The patient was taken to the operating room for a planned robot-assisted partial nephrectomy. Due to the abnormal appearance of the tumor by the urologic surgeon during the case, the decision was made to send a frozen section for identification intra-operatively following tumor resection. This initial specimen was histologically more consistent with a soft tissue tumor, and positive margins were identified after two additional resections, including a margin near the ureter. The decision was made at that time to convert to radical nephrectomy.

The final surgical specimen consisted of five distinct parts. Grossly, the specimen that was labeled as a tumor consisted of a 23-gram, firm, tan-white, irregularly shaped soft tissue fragment measuring 4.6 x 4.3 x 3 cm. There was a normal-appearing red-brown left kidney measuring 4.8 x 3.6 x 0.4 cm attached to the tumor. The cut surfaces of the specimen revealed a tan-white, centrally hemorrhagic tumor with well-demarcated borders that grossly appeared to penetrate the renal parenchyma.

**Figure 2:**
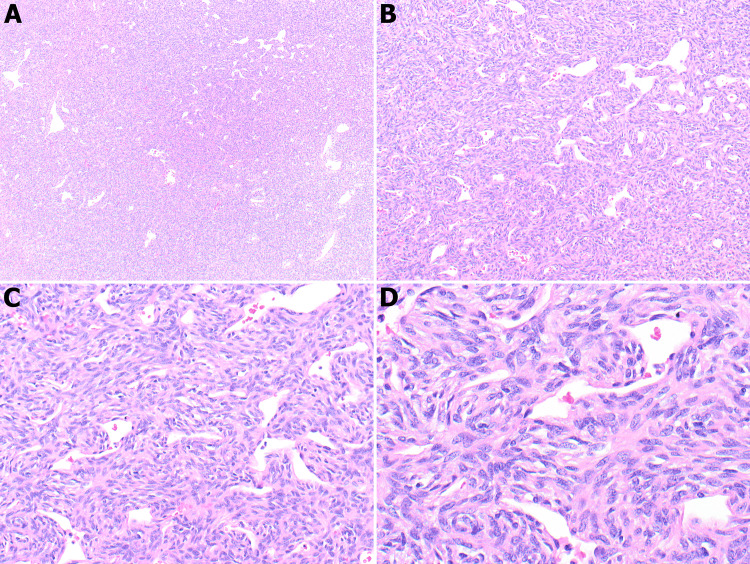
Low and medium power views show the tumor is composed of hypercellular spindle cells with abundant hemangiopericytoma and staghorn-like vasculatures (A, B, C; 4X, 10X, 20X). High-power view showing haphazardly arranged spindle to oval neoplastic cells (D, 40X).

Histological examination of the tumor revealed a spindle cell neoplastic process; the cells were arranged haphazardly, spindled to ovoid in shape, exhibiting both hypo- and hypercellular areas. The tumor was very well vascularized, and the blood vessels displayed a hemangiopericytoma-like appearance with a characteristic staghorn-branched appearance with intervening collagenous bands. Mild cellular atypia is evident; no necrosis or mitotic figures were identified (Figure 2).

**Figure 3:**
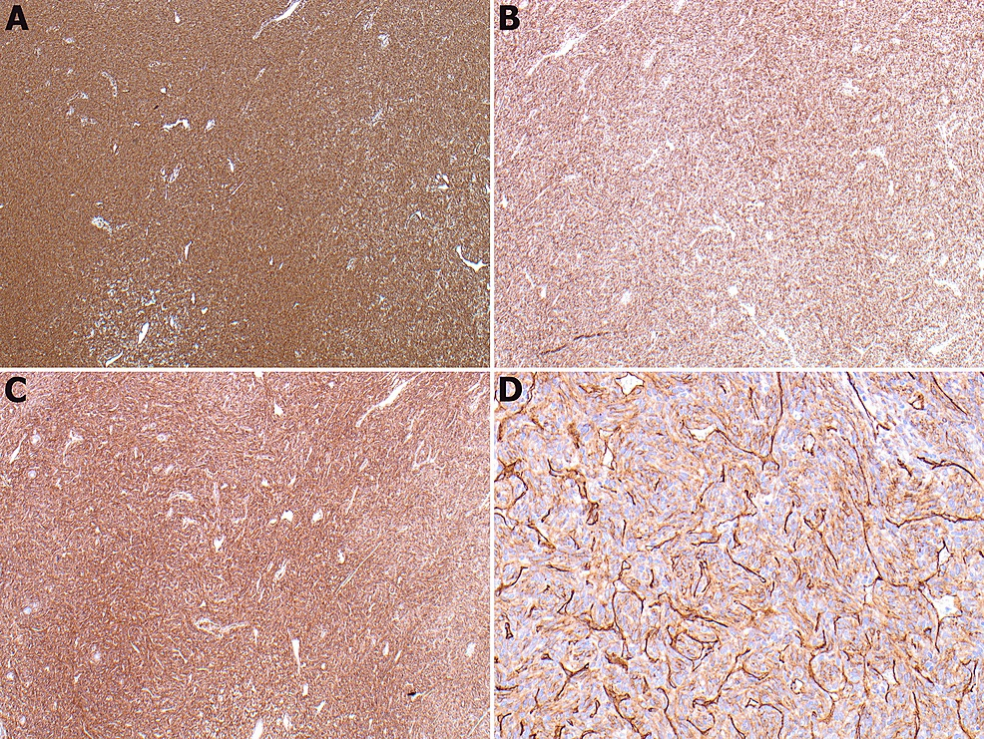
Vimentin, BCL-2, and CD99 show diffuse and strong expression in tumor cells (A, B, C; 4X). CD34 is positive in blood vessels and negative in tumor cells (D, 10X).

Multiple immunohistochemistry stains were performed using antibodies against vimentin, Bcl-2, CD99, and CD34. Vimentin, Bcl-2, and CD99 are strongly and diffusely positive in the neoplastic cells. CD34 is positive in the endothelial cells of the blood vessels and negative in the neoplastic cells (Figure 3).

**Figure 4:**
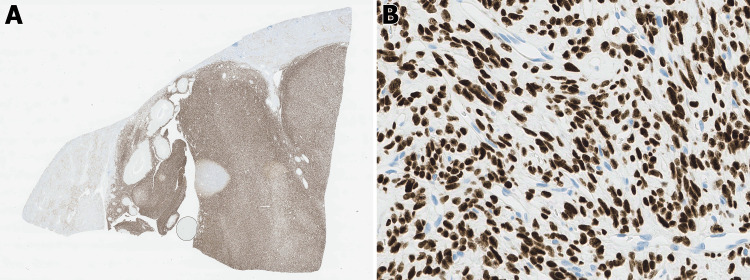
Diffuse and strong expression of STAT6 is diagnostic for SFT-K (A, 4X, B, 20X).

**Figure 5:**
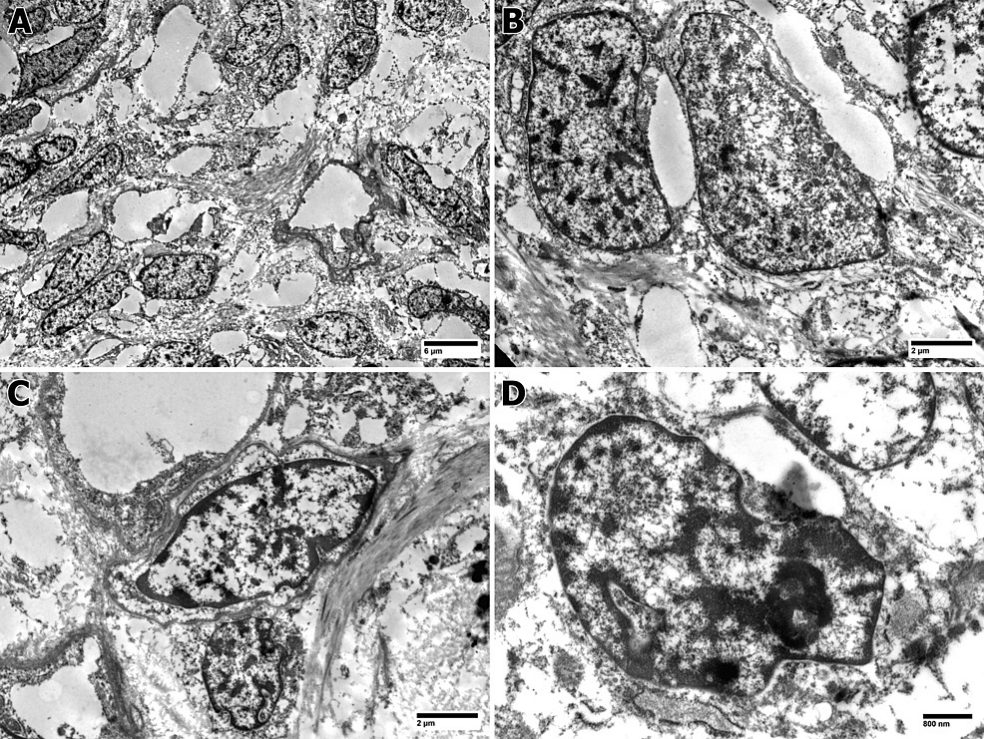
A: Low-power view of neoplasm shows the vessel surrounded by neoplastic cells embedded in an edematous, myxoid stroma (AX600). B, C: Typical cells in the neoplasm exhibit a dilated, rough endoplasmic reticulum, a characteristic feature of fibroblastic cells (BX2000, CX2000). D: High-power view of neoplastic cells. Note the intimate association with collagen fibers in the stroma (DX4000).

**Figure 6:**
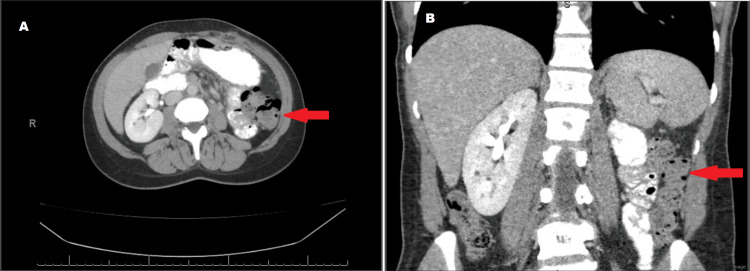
Post-operative cross-axial images (A, B) show expected changes after left nephrectomy without evidence of metastatic disease.

The spindle cells show diffuse and strong nuclear expression of STAT6, compatible with the presence of NAB 2-STAT6 fusion and diagnostic of a solitary fibrous tumor (Figure 4). Electron microscopy was also performed on paraffin embedded formalin fixed (PEFF) tissue block, and findings are shown in Figure 5. The patient was followed by urology, and a two-month follow-up CT scan shows expected post-operative changes with surgical absence of the left kidney. No residual or metastatic disease was identified (Figure 6).

## Discussion

Solitary fibrous tumors (SFTs), classically thought to be of intra-thoracic origin, are rare mesenchymal tumors that can arise virtually anywhere in the body. [1] The Washington Manual of Surgical Pathology by Pfeiffer et al. describes the tumor as a "typically well-circumscribed, nonencapsulated, firm, white mass" with an "average size of 5 to 10 cm” [2]. SFTs infrequently affect the kidneys. Often an incidental finding, clinical symptoms can include flank pain and hematuria [1]. Imaging has limited utility beyond the evaluation of the extension and recurrence of renal tumors. As a result, renal SFTs are usually diagnosed and treated as renal cell carcinoma (RCC) [3].

Microscopic examination of SFTs reveals a hypercellular stroma with bland, plump to spindle-shaped cells and a "pattern-less" architecture with interspersed gaping, branching blood vessels (also known as staghorn vessels). The vascular pattern is reminiscent of hemangiopericytomatous lesions [4]. In fact, according to one case report, SFTs may demonstrate a focal hemangiopericytoma-like growth pattern [2]. The stroma is typically collagenous with focal myxoid areas, and cellularity within a single tumor is variable. The more diffuse cellular tumors were previously classified as hemangiopericytomas [5]. The lipomatous variant of SFT contains mature adipose tissue and may demonstrate giant cells that sometimes form pseudo-vascular spaces, known as the giant cell angiofibroma variant [5].

In 1989, England et al. were the first to propose criteria for malignancy in SFTs [6]. These include the following histological criteria: increased cellularity with crowded/overlapping nuclei; cellular pleomorphism; a mitotic count greater than 4 per 10 high-power fields; the presence of necrosis; and negativity in CD34 and Bcl-2. Clinical criteria, including patient age, large tumor size, and an extra-thoracic location, were also described [2,5].

Immunohistochemistry (IHC) is an essential tool in the diagnosis of SFTs. SFTs are typically positive for CD34, with strong to weak reactivity. Less dependable but potentially beneficial IHC stains include STAT6, Bcl-2, CD99, and vimentin. [1,5] Tumor cells are rarely immunoreactive for desmin and S100. When positive, the staining pattern is usually focal. It has been reported that in malignant cases where CD34 expression may be lost, demonstration of an SYT-SSX gene fusion may establish the renal origin of the tumor [2].

Rendering a diagnosis of renal SFT is complicated by the immunohistochemical overlap between SFT and synovial sarcoma. Synovial sarcomas are negative for desmin, actin, S100 protein, and cytokeratin and positive for vimentin and Bcl-2. Determining the correct entity is aided by CD34 negativity and EMA positivity in synovial sarcomas as compared to benign SFTs [2].

As mentioned earlier, SFTs can demonstrate hemangiopericytoma-like vasculature, a feature that is also shared with synovial sarcomas, as well as malignant peripheral nerve sheath tumor (MPNST), mesenchymal chondrosarcoma, infantile fibrosarcoma, phosphaturic mesenchymal tumor (PMT), thymoma (spindle cell), myopericytoma, deep benign fibrous histiocytoma, and endometrial stromal sarcoma [5]. In fact, according to Fine et al., some lesions previously diagnosed as hemangiopericytoma might be more accurately described as SFT [3]. Sarcomatous RCC should also be included in the differential, given that it is more common than renal SFTs and shares histologic features [2].

There are no standardized guidelines for treating SFT of the kidney; however, a review of the literature suggests that complete resection is associated with a favorable prognosis. Most patients undergo radical nephrectomy, with a few undergoing nephron-sparing surgery [1]. While reports of recurrence after radical nephrectomy are rare, Fine et al. report a case of patient death due to metastatic disease after radical nephrectomy [3].

## Conclusions

This case report sheds light on the rare presentation of a solitary fibrous tumor in the kidney, emphasizing the importance of considering such rare entities in the differential diagnosis of renal lesions. Solitary fibrous tumors of the kidney are uncommon renal neoplasms that present diagnostic and therapeutic challenges due to their rarity and potential morphological overlap with other tumors. Accurate diagnosis and appropriate surgical management are crucial for achieving favorable patient outcomes. Further research and case studies are warranted to enhance our understanding of the pathogenesis, clinical behavior, and optimal management strategies for solitary fibrous tumors, particularly in atypical locations such as the renal pelvis.
